# Successful surgical treatment for nonocclusive mesenteric ischemia of a wide area of the intestine accompanied by gastric conduit necrosis after esophagectomy for esophageal cancer: a case report and review of the literature

**DOI:** 10.1186/s40792-020-00890-1

**Published:** 2020-06-12

**Authors:** Kotaro Miura, Naoshi Kubo, Katsunobu Sakurai, Yutaka Tamamori, Akihiro Murata, Takafumi Nishii, Shintaro Kodai, Akiko Tachimori, Sadatoshi Shimizu, Akishige Kanazawa, Toru Inoue, Yukio Nishiguchi, Kiyoshi Maeda

**Affiliations:** 1 Department of Gastroenterological Surgery, Osaka City General Hospital, 2-13-22 Miyakojimahondori, Miyakojima-ku, Osaka, 534-0021 Japan; 2 Department of Hepato-Biliary Pancreatic Surgery, Osaka City General Hospital, 2-13-22 Miyakojimahondori, Miyakojima-ku, Osaka, 534-0021 Japan; 3 Department of Surgery, Osaka City Juso Hospital, 2-12-27 Nonakakita, Yodogawa-ku, Osaka, 532-0034 Japan

**Keywords:** Mesenteric ischemia, Postoperative complications, Esophagectomy

## Abstract

**Background:**

Nonocclusive mesenteric ischemia (NOMI) has been reported to be a life-threating disease. Gastric conduit necrosis is known as a critical postoperative complication after esophagectomy for esophageal cancer. We encountered a rare case of NOMI of a wide area of the intestine accompanied by gastric conduit necrosis after esophagectomy, which was successfully treated with an emergency operation.

**Case presentation:**

A 67-year-old man presented with dysphagia. He was diagnosed with middle thoracic advanced esophageal cancer. After neoadjuvant chemotherapy, he underwent subtotal esophagectomy with lymphadenectomy and gastric conduit reconstruction. On postoperative day (POD) 2, he had diarrhea, high fever, and low blood pressure, which were treated with catecholamines. Laboratory data revealed acidosis and severe sepsis with multi-organ failure, including the kidneys. Although enhanced computed tomography did not exhibit definite findings of bowel ischemia, upper gastrointestinal endoscopy revealed necrotic mucosal changes in the whole gastric conduit. Therefore, we made a diagnosis of septic shock caused by gastric conduit necrosis and performed an emergency operation. When we explored the abdominal cavity, we found not only gastric conduit necrosis but also intermittent necrotic changes in the intestinal wall from the jejunum to the rectum. Therefore, NOMI was diagnosed. We performed an excision of the gastric conduit and 2 m of the small intestine, as well as total colectomy. After the second operation, prostaglandin E1 was administered intravenously as the treatment for NOMI, and sepsis was improved. On POD 122, he was self-discharged. He died of recurrence of lung metastasis from the esophageal cancer 9 months after the first operation.

**Conclusion:**

When a patient has a critical status, including severe sepsis or severe acidosis, after esophagectomy, we should consider the possibility of NOMI in addition to gastric conduit necrosis and aim to diagnose and treat it immediately with an urgent operation.

## Background

Nonocclusive mesenteric ischemia (NOMI) causes ischemia of the mesentery due to absence of mechanical obstruction of the mesenteric vessels by thrombus or embolism [1–3]. NOMI has been reported to be a life-threating disease. Gastric conduit necrosis is known to be a critical postoperative complication following esophagectomy for esophageal cancer [4, 5]. We herein report a rare case of nonocclusive mesenteric ischemia of a wide area of the intestine accompanied by gastric conduit necrosis following esophagectomy, which was treated successfully with an emergency operation.

## Case presentation

**Fig. 1:**
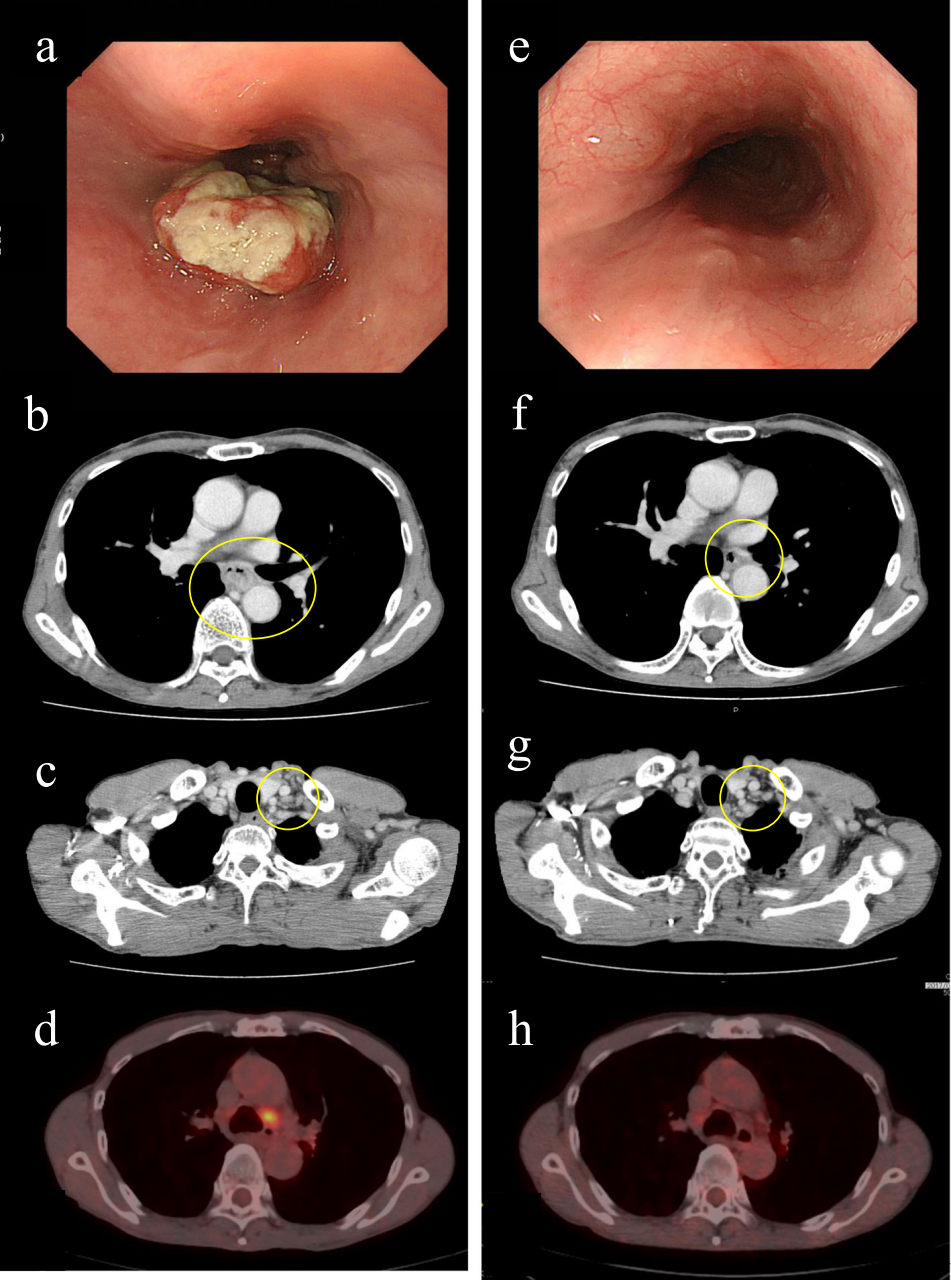
Endoscopy and imaging before neoadjuvant chemotherapy (NAC) for esophageal cancer (**a**–**d**) and the findings after NAC (**e**–**h**). Endoscopy revealed marked shrinkage of the primary tumor (**a**, **e**). Computed tomography also revealed contraction of the primary tumor (indicated by a yellow circle in **b**, **f**). The sizes of the cervical lymph nodes defined as no. 104 by the Japanese Esophageal Society were stable after NAC (indicated by a yellow circle in **c**, **g**). Positron emission tomography also decreased uptake in the primary tumor (**d**, **h**)

A 67-year-old Japanese man presented with dysphagia. He had a medical history of exploratory laparotomy for ileus but no history of anticoagulant medication. Upper gastrointestinal (GI) endoscopy revealed type III esophageal cancer in the middle thoracic esophagus (Fig. 1a). An upper GI series revealed an irregular stricture in the middle thoracic esophagus. Computed tomography (CT) revealed a primary tumor and swollen cervical and mediastinal lymph nodes (Fig. 1b, c). Fluorodeoxyglucose-positron emission tomography (FDG-PET) also revealed a primary tumor in the esophagus (Fig. 1d) and swollen lymph nodes in the mediastinum with abnormal uptake.

As a result, he was diagnosed with middle thoracic esophageal cancer (squamous cell carcinoma) and T3N3M0 in clinical stage III according to the criteria of the Japan Esophageal Society. He was treated with combination chemotherapy of docetaxel, cisplatin, and fluorouracil as neoadjuvant therapy. Chemotherapy for 2 months resulted in shrinkage of the primary tumor of the esophagus and lymph nodes in the mediastinum (Fig. 1e–g). FDG-PET revealed decreased uptake in the primary tumor and lymph nodes in the mediastinum (Fig. 1h).

After chemotherapy, he underwent subtotal esophagectomy with three-field lymphadenectomy and gastric conduit reconstruction through the retrosternal route by thoracoscopy and laparotomy. Postoperative care was performed under mechanical ventilation in the intensive care unit (ICU). On postoperative day (POD) 1, mechanical ventilation was terminated because of a good respiratory condition. However, he suffered from dyspnea and severe acidosis and chest X-ray examination revealed right pneumothorax, which was observed 5 h after extubation. Furthermore, air leakage was observed from the water seal chamber of the chest drain system. We thought that the respiratory disorder was caused by the right pneumothorax. Therefore, mechanical ventilation was started again. After that, it became clear that the cause of the pneumothorax was the air sucked from his drain insertion site. A few sutures were added at his drain insertion site. And we decided to continue his treatment under mechanical ventilation until the respiratory problems were improved.

**Fig. 2:**
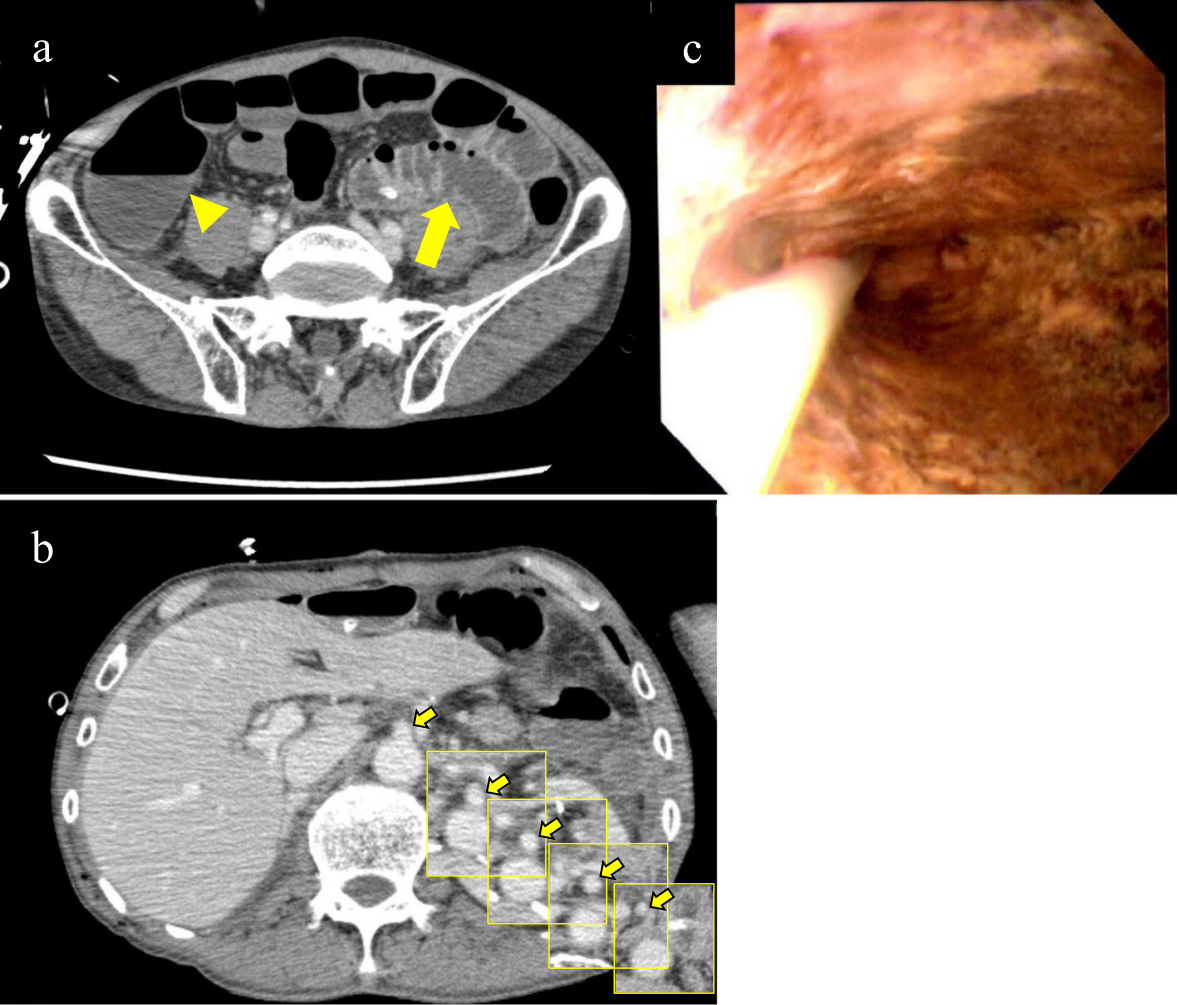
Computed tomography (CT) and endoscopy at postoperative day 2 following esophagectomy. **a** CT revealed dilation of the small intestine (arrow) and large intestine (arrow head). We interpreted this finding as enteritis at first. **b** These images show slices of the superior mesenteric artery (SMA). CT revealed that there was no thrombus in the SMA (arrow). **c** Upper gastrointestinal endoscopy revealed ischemic changes in the mucosa over the entire circumference of the gastric tube from the anastomotic site to the antrum

**Fig. 3:**
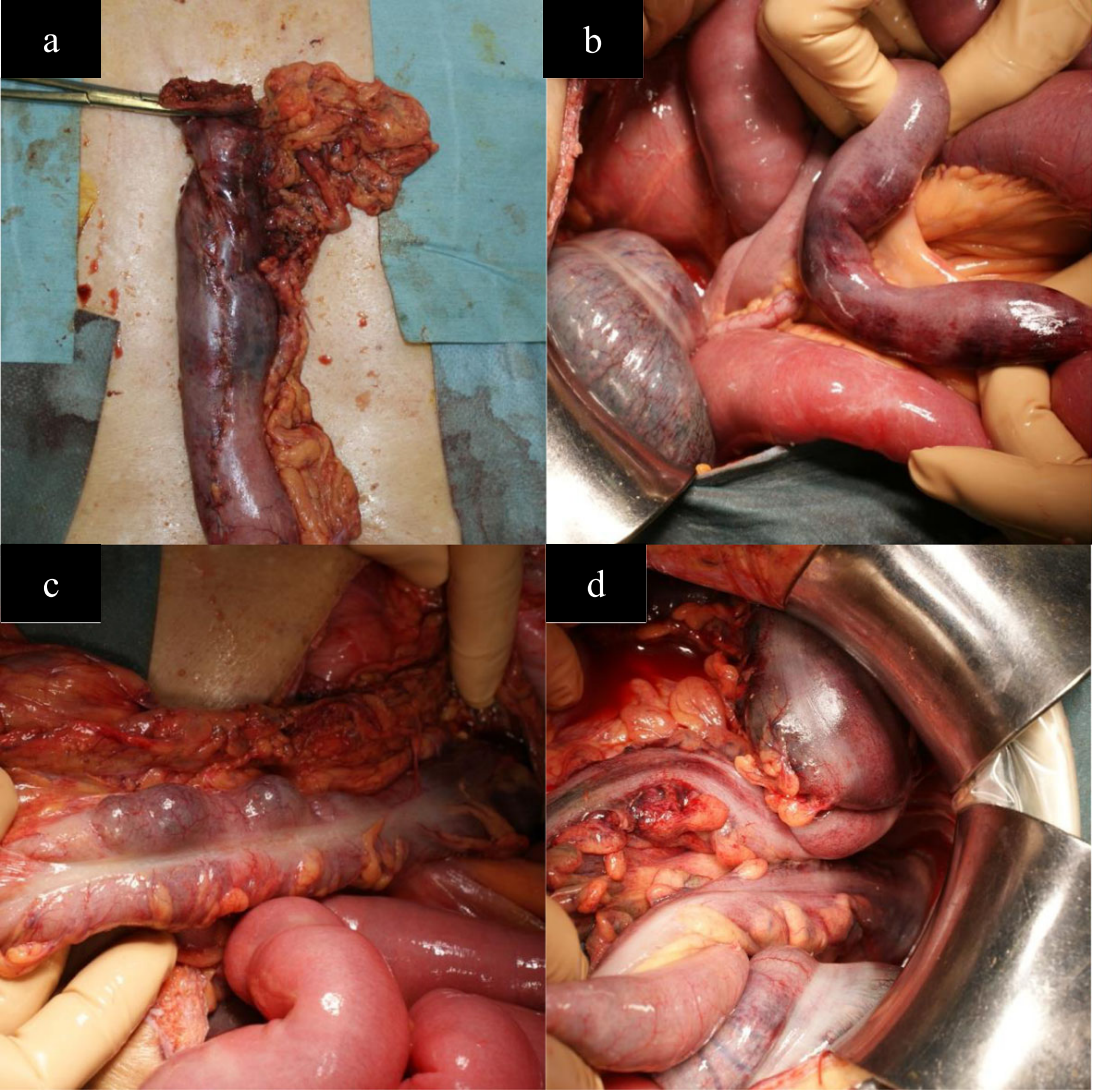
Surgical findings at the second operation. **a** The color of the gastric conduit changed to purple, except for the antrum region. **b** The small intestine exhibited segmental ischemic changes. **c** Almost all of the colon, except for a part of the transverse colon, also exhibited necrotic changes. **d** Rectum near the peritoneal reflection was not preserved because of the ischemic change

**Fig. 4:**
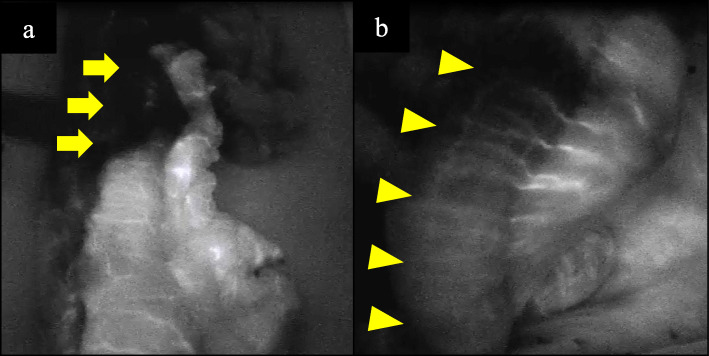
Estimation of mesenteric blood flow by indocyanine green fluorescence. **a** The upper gastric tube was not contrasted (arrow). We decided to perform an entire resection of the gastric tube due to this finding in addition to macroscopic and endoscopic findings. **b** A large part of the small intestine was also not contrasted (arrow head). The segmentally non-contrasted small intestine was resected even if the region can be preserved due to the macroscopic color of the serosa

**Fig. 5:**
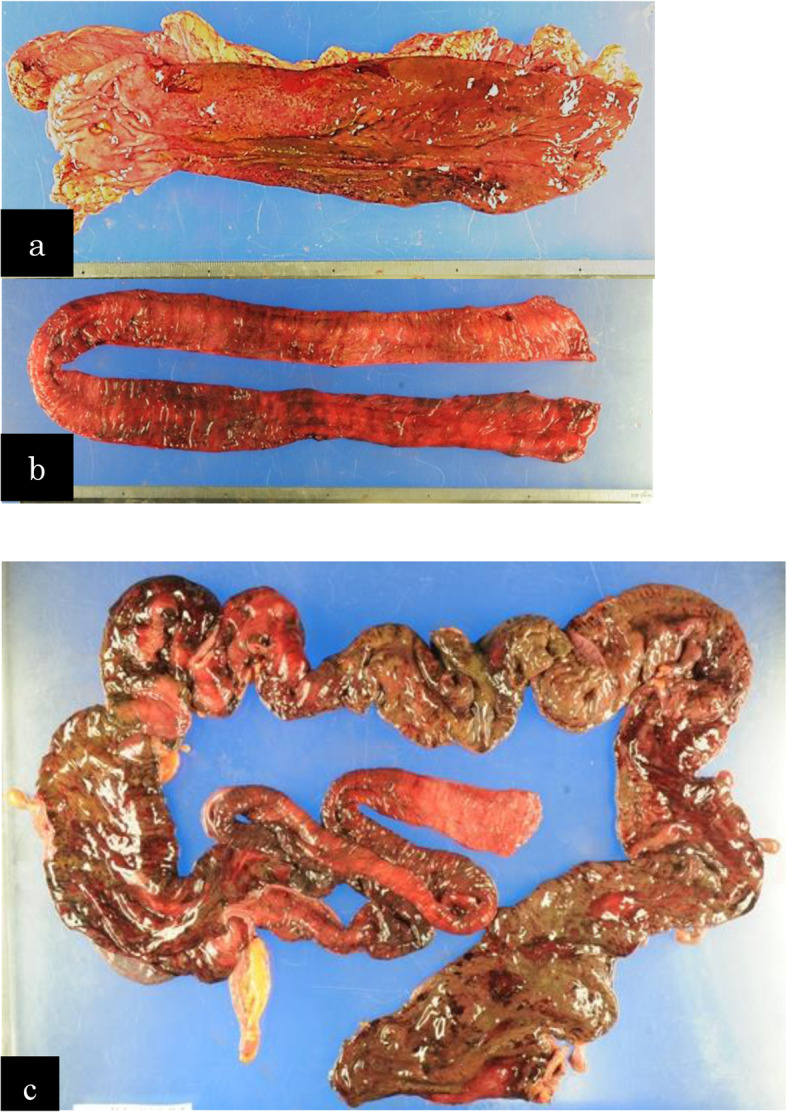
Macroscopic findings of the resected specimen. **a** Gastric tube. Macroscopic color was worse, especially in the upper region. **b** Additional resected small intestine. **c** Ileum and large intestine. Almost all of the large intestines exhibited ischemic changes

**Fig. 6:**
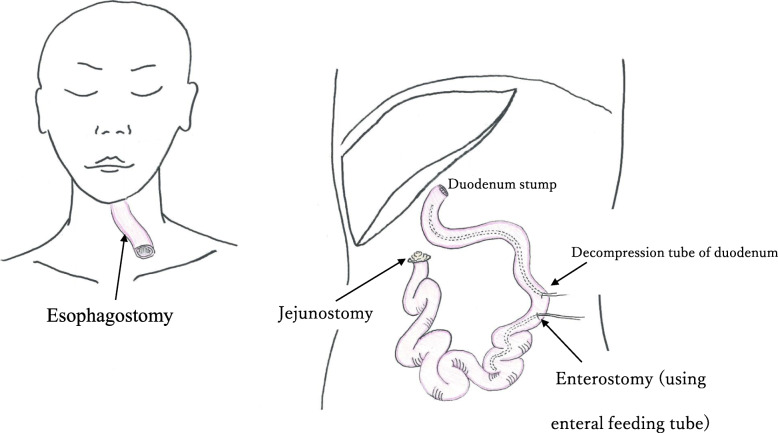
An outline of the reconstruction in the second operation. Esophagostomy, jejunostomy, and enterostomy for nutrition were performed. The length of the remnant jejunum was 1 m and 80 cm

On POD 2, the pneumothorax was improved. However, he had diarrhea, fever, and low blood pressure, which were treated with catecholamines. At the time, his abdomen was flat and soft. Laboratory findings revealed worsened inflammation with a decline in the white blood cell count, deviated enzyme levels, renal failure, and acidosis (Table 1). All things considered, severe sepsis with multi-organ failure was suspected. Enhanced CT revealed bowel dilation with increases in the intestinal fluid, including the gastric conduit (Fig. 2a). There were no findings of arterial thrombus, including in the superior mesenteric artery (Fig. 2b). Upper GI endoscopy revealed necrotic mucosal changes in the whole gastric conduit (Fig. 2c). Therefore, septic shock caused by gastric conduit necrosis was diagnosed, and an emergency operation was planned. When we explored the abdominal cavity, we found not only gastric conduit necrosis but also intermittent necrotic changes in the intestinal wall from the jejunum to the rectum (Fig. 3). Based on the findings, we considered a diagnosis of NOMI. The extent of excision was determined first by macroscopy. The non-enhanced lesion by indocyanine green (ICG) fluorescence in the small intestine was excised additionally (Fig. 4). As a result, we performed an excision of the gastric conduit and 2 m of the small intestine, which was followed by a total colectomy (Fig. 5). After resection, the length of the remnant jejunum was 1 m and 80 cm. We considered re-reconstruction after the removal of the gastric conduit. However, we decided that re-reconstruction could not be made because the surgical stress of re-reconstruction was intolerable considering his critical status. Esophagostomy, jejunostomy, and enterostomy for nutrition were performed (Fig. 6).

**Table 1:** Laboratory findings and arterial blood gas findings on postoperative day 2 after esophagectomy

WBC	2530/μl
RBC	3.04 × 10^6^/μl
Hb	10.1 g/dl
Plt	147 × 10^3^/μl
AST	159 U/l
ALT	57 U/l
LDH	556 U/l
CK	2064 U/l
BUN	46.3 mg/dl
Cre	2.02 mg/dl
CRP	26.6 mg/dl
pH	7.225
PaO_2_	95.9 mmHg
PaCO_2_	53.5 mmHg
PaHCO_3_	21.3 mmol/l
Base excess	− 5.8 mmol/l
Lactate	13 mg/dl

*WBC* white blood cell, *RBC* red blood cell, *Hb* hemoglobin, *Plt* platelet, *AST* aspartate aminotransferase, *ALT* alanine aminotransferase, *LDH* lactate dehydrogenase, *CK* creatine kinase, *BUN* blood urea nitrogen, *Cre* creatinine, *CRP* C-reactive protein

The pathological findings revealed shedding of the epithelium, hemorrhagic necrosis of the mucosa, capillary dilation of the submucosa, and transmural necrobiosis in the gastric conduit, small intestine, and large intestine, which were compatible with NOMI. Almost all of the lesions had no thrombosis in the resected specimens. After the second operation, sepsis was improved. Furthermore, 0.01 μg/kg/min of prostaglandin E1 had been administered intravenously for a week, and enhanced CT findings revealed no ischemic changes in the residual small intestine after the second operation. On POD 16, the patient was discharged from the ICU. On POD 122, he was self-discharged from our hospital after a long period of nutritional support, including total parenteral nutrition and enteral nutrition.

The pathological diagnosis of esophageal cancer was squamous cell carcinoma and pT3N1M0 stage III. On POD 64, follow-up CT revealed the recurrence of esophageal cancer at the para-aortic lymph node. Although, we considered re-reconstruction of the esophagus, we abandoned this strategy, because his condition could not tolerate surgical stress, and recurrence of esophageal cancer was confirmed. He died of the recurrence of lung metastasis from esophageal cancer 9 months after the operation.

## Discussion

We report a rare case of nonocclusive mesenteric ischemia of a wide area of the intestine accompanied by gastric conduit necrosis following esophagectomy, which was treated successfully with an emergency operation.

Previously, to our knowledge, NOMI following esophagectomy was reported in only 12 cases (Table 2 [6–12]).

**Table 2:** Twelve cases of bowel necrosis or NOMI after esophagectomy

Author	Age	Sex	Comorbidity	Diagnosis	Symptoms after IS	Abnormal findings after IS	CT findings after IS	Treatment	The extent of ischemic change	Outcome
Lawlor [6]	65	F	ND	Barrett’s esophagus and severe dysplasia	Abdominal distension	Respiratory and renal failures	ND	Intestinal resection	10 cm proximal to the jejunostomy and extending to the cecum	Survival
	57	M	ND	EC	Abdominal distension	Fever, hypotension, respiratory and renal failures	Pneumatosis intestinalis	Intestinal resection	Small intestine distal to jejunostomy	Survival
Hokamura [7]	71	M	ND	EC	Abdominal pain	Base excess: − 5	ND	Intestinal resection	Almost the entire small intestine	Dead
	70	M	ND	EC	Abdominal pain	Base excess: − 8	ND	Intestinal resection	Almost the entire small intestine and colon	Dead
	75	M	ND	EC	ND	Base excess: − 7	ND	Intestinal resection	Almost the entire small intestine	Dead
Melis [8]	54	F	Hypertension	EC	Abdominal distension and discomfort	Respiratory and renal failures	Mild ascites and distended loops of small and large intestines	Intestinal resection	Jejunostomy and extending distally for about 40 cm	Dead
Qureshi [9]	58	M	Myocardial infarction	EC	Abdominal distension	Supraventricular tachycardia	Mediastinal collection	Laparotomy	Congested gastric tube with sloughing at the anastomotic site, entire small and large intestines	Dead
Sethuraman [10]	60	ND	Hyperlipidemia	EC	Abdominal distension and pain	Increased nasogastric tube output, hypotension, respiratory and renal failures	ND	Intestinal resection	Distal to the jejunostomy	Survival
	74	ND	Hypertension, diabetes	EC	ND	Leukocytosis, fever, abdominal compartment syndrome	ND	Intestinal resection	Distal to the jejunostomy	Survival
Irie [11]	62	M	Hypertension	EC	Abdominal pain	Pyrexia, lowered renal function, elevated CRP value	Pneumatosis intestinalis	Open abdominal management	50–170 cm proximal to the ileum	Survival
Kurita [12]	75	M	None	EC	Diarrhea, abdominal pain and distension	Fever, bloody drainage through gastrostomy, leukocytosis, elevated CRP value	Hepatic portal venous gas, dilated digestive tract with pneumatosis intestinalis, ascites	Papaverine	ND	Survival
	68	M	Diabetes, atrial fibrillation	EC	Diarrhea, abdominal pain and distension	Bloody drainage through jejunostomy, leukocytosis, elevated CRP value	Hepatic portal venous gas, dilated digestive tract with pneumatosis intestinalis, ascites	Papaverine	ND	Survival

*NOMI* nonocclusive mesenteric ischemia, *IS* initial surgery, *ND* no description, *EC* esophageal cancer

As presented in Table 2, 10 patients complained of abdominal distension or pain a few days following esophagectomy. As objective findings, acidosis and organ failure were observed in seven cases. CT findings revealed pneumatosis intestinalis in four cases. However, there were no common clinical findings in all 12 cases. Generally, there were no common and specific findings suggesting NOMI [3]. Therefore, a definite diagnosis of NOMI is difficult, and the mortality rate remains high [2]. However, recent reports revealed that there were symptoms and laboratory findings suggesting NOMI [2, 13]. For example, abdominal distension, which cannot be explained, or digestive tract hemorrhage might be one of the symptoms of NOMI. However, the symptoms are unclear in many critically ill ventilated patients. In addition, a patient’s critical status, including new onset of organ failure and an increase in vasoconstrictors, should be suspected as a symptom of mesenteric ischemia [13]. In CT findings, a contraction of the bowel loops, bowel dilation, or a fluid-filled bowel and intestinal pneumatosis could be observed in various phases of NOMI [2, 3]. Especially, bowel dilation in NOMI is observed in the colon, and the finding could be useful for differential diagnosis between NOMI and other types of acute mesenteric ischemia [2]. In our report, we could not evaluate the degree of abdominal pain because of sedation for mechanical ventilation. Diarrhea, severe acidosis, and renal failure were observed, as in previous reports. In a sedated patient, these observational findings might be key to diagnosing NOMI. Retrospectively, the CT findings of our patient revealed bowel dilation and fluid-filled bowel, which are suggestive of NOMI. When those findings, suggesting ischemic change of the intestine, are observed following esophagectomy, we should consider the possibility of NOMI and aim to diagnose NOMI rapidly with various modalities.

Regarding treatment of the previous 12 cases of NOMI following esophagectomy, an emergency laparotomy was performed in 10 cases, and nonsurgical treatments, including intra-arterial or intravenous infusion of papaverine, were performed in two. Previous reports revealed that management of NOMI was initially needed improvement of hemodynamics and exclusion of vasoconstrictors [1, 13]. Additional treatments, including anticoagulation, vasodilators, and surgery, should be administered as needed [1, 13]. In exploratory laparotomy, complete resection of the intestine with necrotic lesion is required [13]. Although there were a few case reports in which nonsurgical treatment was selected and was successful, we infer that the first choice of NOMI treatment should generally be surgery. Nonsurgical treatment needs an interventional radiologist and a treatment unit that can respond to rapid worsening of the pathophysiology of NOMI, and therefore, this treatment could not be applied in all institutions. As for our patient, since we suspected gastric conduit necrosis based on the result of the upper GI endoscopy, an exploratory laparotomy was performed. As a result, NOMI could be eventfully diagnosed during the operation. The extent of resection of the intestine was adequately selected with macroscopy and an ICG fluorescence technique, similar to a previous report [11, 14]. As for our patient, the ICG fluorescence technique was beneficial because the ischemic lesion could be identified more objectively than with macroscopic findings.

Among the reported cases, 5 of 12 patients died during the early postoperative periods. The widespread intestine was resected in many of the terminal cases. Resection of a large part of the small and large intestines was also required in our patient. Nevertheless, our patient was alive after the surgery for NOMI. The reasons might be that an adequate area was excised and that the necrotic lesion of the small intestine was narrower than that in the previous report.

Furthermore, only in our patient, NOMI was accompanied by gastric conduit necrosis following esophagectomy. To our knowledge, this phenomenon was not reported in other cases. Gastric conduit necrosis is also a rare critical complication following esophagectomy [4, 5]. In addition, cardiac failure, postoperative hypotension, use of vasoconstrictor, etc. were suggested as risk factors for gastric conduit necrosis [5]. These factors were similar to those for NOMI [1, 3]. Because our patient had these risk factors following esophagectomy, NOMI and gastric conduit necrosis might occur simultaneously. Although both critical complications occurred, our patient survived because of rapid examinations and intensive care with surgery.

## Conclusion

We encountered a case of NOMI accompanied by gastric conduit necrosis following esophagectomy, and the patient was treated with adequate resection of the necrotic digestive tract and provided with intensive care. When a patient is in critical status, including severe sepsis or severe acidosis, following esophagectomy, we should consider the possibility of NOMI in addition to gastric conduit necrosis and aim to diagnose and treat it immediately with an emergency operation.

## Data Availability

All data generated during this report are included in this published article.

## Abbreviations

NOMI: Nonocclusive mesenteric ischemia
GI: Gastrointestinal
CT: Computed tomography
FDG-PET: Fluorodeoxyglucose-positron emission tomography
ICU: Intensive care unit
POD: Postoperative day
ICG: Indocyanine green
